# Antigen-Specific T-Cell Activation Independently of the MHC: Chimeric Antigen Receptor-Redirected T Cells

**DOI:** 10.3389/fimmu.2013.00371

**Published:** 2013-11-11

**Authors:** Markus Chmielewski, Andreas A. Hombach, Hinrich Abken

**Affiliations:** ^1^Center for Molecular Medicine Cologne, University of Cologne, Cologne, Germany; ^2^Department I Internal Medicine, University Hospital Cologne, Cologne, Germany

**Keywords:** chimeric antigen receptor, T-cell receptor, adoptive cell therapy, antibody, antigen-presenting cell

## Abstract

Adoptive T-cell therapy has recently shown promise in initiating a lasting anti-tumor response with spectacular therapeutic success in some cases. Specific T-cell therapy, however, is limited since a number of cancer cells are not recognized by T cells due to various mechanisms including the limited availability of tumor-specific T cells and deficiencies in antigen processing or major histocompatibility complex (MHC) expression of cancer cells. To make adoptive cell therapy applicable for the broad variety of cancer entities, patient’s T cells are engineered *ex vivo* with pre-defined specificity by a recombinant chimeric antigen receptor (CAR) which consists in the extracellular part of an antibody-derived domain for binding with a “tumor-associated antigen” and in the intracellular part of a T-cell receptor (TCR)-derived signaling moiety for T-cell activation. The specificity of CAR-mediated T-cell recognition is defined by the antibody domain, is independent of MHC presentation and can be extended to any target for which an antibody is available. We discuss the advantages and limitations of MHC-independent T-cell targeting by an engineered CAR in comparison to TCR modified T cells and the impact of the CAR activation threshold on redirected T-cell activation. Finally we review most significant progress recently made in early stage clinical trials to treat cancer.

## Tumor-Specific T Cells for Adoptive Cell Therapy

Experimental and clinical evidences indicate that the immune system is capable of identifying and destroying cancer cells in a specific fashion; tumor-infiltrating lymphocytes (TILs), expanded *ex vivo* and re-administered to the patient, exhibit a powerful anti-tumor response and induce an acute inflammatory reaction which attracts a second, antigen-independent wave of immune cell invasion into the same lesion. Adoptive TIL therapy has shown some success in the treatment of chemotherapy resistant melanoma, even in advanced stages of the disease (1). The procedure, however, is technically challenging since it involves the isolation of T cells from melanoma biopsies and their amplification *ex vivo* to therapeutic numbers; not every melanoma biopsy provides TILs and allows sufficient expansion. Moreover, the range of TIL bearing malignant lesions, apart from melanoma, is small limiting the application of the strategy to a broad variety of cancer entities.

The implementation of redirected T cells in cancer therapy is based on engineering T cells with pre-defined specificity to target virtually every cancer cell and on the production of engineered T cells in therapeutic numbers. To provide specificity peripheral blood T lymphocytes were *ex vivo* engineered with a recombinant T-cell receptor (TCR) of known specificity which recognizes cognate peptide-loaded major histocompatibility complexes (pMHC) of a so-called tumor-associated antigen (TAA). Such TCR engineered T cells showed promise in clinical trials (1, 2). Some conceptual deficits, however, limit the broad application of TCR engineered T cells including the HLA restriction, the dependency on adequate major histocompatibility complex (MHC) expression by tumor cells, the limited number of peptide-MHC complexes identified so far which can be used for screening and the potential mispairing with the endogenous TCR producing novel, unforeseen specificities which might induce severe auto-immunity after adoptive transfer (3).

Whereas the T-cell therapy using *ex vivo* expanded patients’ TILs leads to significant clinical effect in patients with metastatic melanoma (1), difficulties are arising when engineering T cells with a recombinant TCR, in particular when non-immunogenic tumor-associated self-antigens are targeted (4). In a pre-clinical tumor model the treatment with TCR engineered T cells alone was without effect while the combination of vaccination with TCR modified T-cell transfer was synergistic.

**Figure 1:**
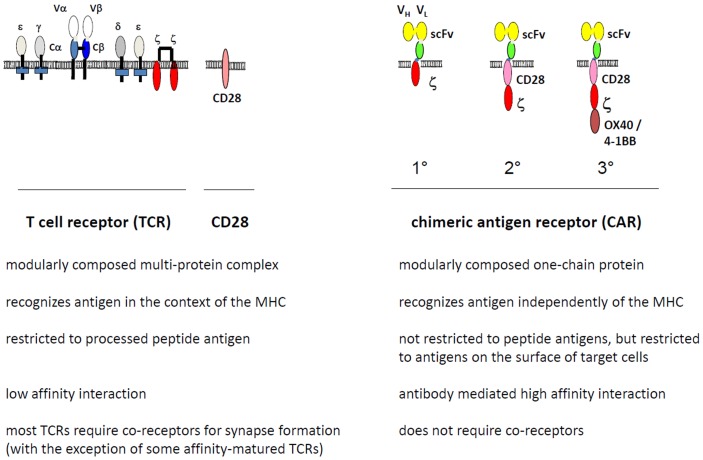
**Modular composition of the chimeric antigen receptor (CAR) compared to the T-cell receptor (TCR)**. The TCR binds to cognate peptide-loaded MHC (pMHC) by the TCR α and β chains, forms the immunological synapse by clustering accessory components including CD3ζ and CD28, and initiates the downstream signaling pathway for T-cell activation through phosphorylation of the CD3ζ ITAM motives. The CAR, in contrast, is composed of one polypeptide chain; the extracellular single chain fragment of variable region (scFv) antibody domain binds to the target antigen in a MHC-independent fashion. Upon CAR clustering, the intracellular CD3ζ chain, with or without costimulation through members of the CD28 family, initiates the downstream signaling for T-cell activation. Co-receptors may modulate CAR activity. In contrast to a first generation (1°) CAR, second (2°), and third (3°) generation CARs harbor in addition one or more costimulatory moieties in their intracellular part.

In this situation, Zelig Eshhar, Weizmann Institute, proposed to redirect T cells by a recombinant receptor molecule, a chimeric antigen receptor (CAR), which in the extracellular part consists of an antibody with pre-defined binding specificity to a broad variety of targets and in the intracellular part of a T-cell activation domain (5). Such CAR modified T cells became generally known as “T-bodies” (5). In contrast to the TCR, the archetypical CAR is composed of one polypeptide chain (Figure 1). The binding domain is mostly a recombinant antibody in the single chain format consisting of the variable domain of the heavy and light chain linked by a short synthetic peptide (scFv). The extracellular part of a receptor molecule, for instance the NK cell-derived NKG2D ligands (6) and the surface NKp-30 (7) receptor, were also successfully integrated into the conventional CAR structure instead of the classical antibody-derived binding domain. The CAR intracellular signaling domain is preferentially derived from the CD3 ζ-chain of the TCR/CD3 complex or, alternatively, from the γ-chain of the high affinity IgE Fc receptor-I (FcϵRI). Binding with cognate antigen on the tumor cell surface results in CAR clustering on the engineered T-cell with the consequence that the immunoreceptor tyrosine-based activation motifs (ITAMs) of the signaling moiety become phosphorylated and initiate a downstream signaling cascade which finally induces T-cell amplification, cytokine secretion, and cytolytic activity of the CAR T-cell toward the cognate tumor cell.

CAR T cells overcome some limitations of the TCR-based strategy by targeting cells in an MHC- and dendritic cell (DC)-independent fashion. The properties of a CAR and TCR differ substantially in respect to target binding and subsequent T-cell activation. For instance these are in particular the TCR avidity for a given pMHC, the number of MHC molecules, the availability of co-receptors and the moderate TCR affinity for the cognate MHC peptide complex compared to the high affinity of the antibody in a CAR. On the one hand the use of antibody-based CAR T cells enables the targeting of antigens of different composition and structure such as peptides, carbohydrates, or inorganic compounds, and on the other hand, the TCR recognizes peptide antigens exclusively in the context of the particular MHC and thereby faces a limited variability. TCRs are moreover inherently cross-reactive toward endogenous antigens (8). The potential CAR targets thereby far outnumber their MHC presented counterparts which can be recognized by TCR modified T cells. In this report we review some advantages and limitations of MHC-independent target recognition by CAR T cells and review most significant progress recently made in early stage clinical trials to treat cancer.

## The CAR Strategy: Antibody-Mediated, MHC-Independent Antigen Recognition by Engineered T Cells

The design of the antibody-derived CAR differs in several major features from the TCR which physiologically mediates target recognition by T cells (Figure 1). By using an antibody for binding, T cells gain antibody-defined specificity: (i) T cells without CAR or equipped with a CAR of different specificity are not activated by the target cells; (ii) the cognate antigen needs to be on the cell surface to trigger CAR T-cell activation, intracellular antigens are not recognized by the CAR; and (iii) CAR-mediated T-cell activation can be specifically blocked by an antibody directed toward the CAR binding domain (9).

As a consequence of using an antibody for binding, CARs can redirect T cells toward targets of any chemical composition or conformation as far as an antibody is available. Indeed, CARs were engineered which target T cells toward carbohydrate antigens like CA19-9 (10–12). The TCR, in contrast, is restricted to the recognition of specific peptides presented by the particular MHC. Antibody-mediated target recognition by CARs, however, does not exclude targeting MHC presented antigens. Using an antibody which recognizes NY-Eso-1 peptide (157–165) in the context of HLA-A*0201, Stewart-Jones et al. engineered a CAR recognizing the MHC presented peptide analog SLLMWITQV (13). The antibody domain used for CAR targeting was optimized by modification of the individual amino acids which interact between the antibody and the peptide providing an antibody with 20-fold improved affinity, exceeding the affinity of the respective TCR by about 1000-fold. The high affinity antibody when engineered as recombinant CAR on T cells conferred specific killing of HLA-A*0201/NY-ESO-1_157–165_ target cells as do T cells modified with the corresponding TCR.

## Both CD8^+^ and CD4^+^ T Cells can be Redirected in a MHC-Independent Fashion

By bypassing MHC class I and class II restriction by an antibody-derived binding domain, CAR engineered T cells of both CD8^+^ and CD4^+^ subsets can be recruited for redirected target cell recognition (9, 14, 15). Equipped with a CAR, CD4^+^ T cells showed as cytolytic as do CD8^+^ T cells toward CAR-defined target cells. While human CD8^+^ T cells predominantly use two pathways in executing cytolysis, i.e., perforin and granzyme exocytosis and to some extend death receptor signaling via Fas/Fas-ligand (Fas-L) or TNF/TNF-receptor (TNF-R) (16), the mechanism of CAR-mediated lysis by redirected CD4^+^ T cells was a matter of debate for some time. Investigations utilizing mutant and knock-out mice suggest that MHC class II restricted cytolysis by murine CD4^+^ T cells is predominantly mediated by the death receptor system (17, 18) which is in contrast to MHC class I restricted cytolysis by CD8^+^ CTLs relying mainly on perforin and granzymes. Accordingly, murine CD8^+^ T cells engineered with a CAR lyse Fas resistant target cells whereas CD4^+^ T lymphocytes do not (19). In contrast to murine cells, CAR redirected human T cells mediate cytolysis predominantly by granzyme/perforin which can be executed independently of Fas or TNF-α signaling (14). The extent in redirected cytolysis correlates with the amount of cytolytic effector molecules; CAR CD4^+^ T cells which harbor about half amounts of perforin and granzyme B required about twice the number of effector cells to achieve the same cytolytic efficacy compared to CAR redirected CD8^+^ T cells. CAR CD4^+^ T cells rapidly lyse their targets in a short term *in vitro* cytotoxicity assay as do engineered CD8^+^ T cells which is in accordance to a perforin mediated process whereas death receptor signaling induces cytolysis of the delayed type. CAR engineered CD4^+^ T cells lyse both Fas- and TNF-resistant target cells. The observation is in accordance to a report that non-modified human CD4^+^ T cells execute cytolysis predominantly by granule exocytosis and not by the Fas/Fas-L system (20). In contrast to CAR modified cells, CD4^+^ cells engineered with a MHC class I restricted TCR were reported to lyse exclusively those target cells that are susceptible for death receptor signaling (21). Both studies, however, differ in several issues including the use of a MHC class I-dependent TCR vs. a MHC-independent CAR for redirecting T cells. As a consequence for adoptive cell therapy, CAR engineered patient’s CD4^+^ T cells can efficiently provide help upon CAR-mediated activation and can eliminate tumor cells in a direct fashion and independently from MHC class II restriction.

## “Affinity Ceiling” of Antibody-Mediated CAR T-Cell Activation

CAR-mediated T-cell activation is thought to depend on and to increase with the binding affinity to cognate antigen; however, the interaction is likely of higher affinity than binding of the physiological TCR to peptide loaded MHC. Two studies addressed in detail the situation (22, 23). The Chmielewski study (22) made use of a panel of CARs of the same backbone and same epitope specificity but with different binding affinities. The affinities were in the broad range of 10^−7^–10^−11^ M and were obtained upon mutation of the parental antibody while preserving the binding specificity. CAR T-cell activation correlated with the affinity of the antibody binding domain when the target antigen is present in an immobilized fashion coated onto surfaces. In contrast, when the cognate antigen is present on the surface of the target cell, the CAR-mediated cytotoxic effect on target cells and the release of IFN-γ and IL-2 did not increase with the binding affinity above threshold, which was in that example about *K*_D_ = 10^−8^ M. While the conditions that define the activation threshold, however, are so far not understood on the molecular level, the study makes clear that furthermore increase in affinity above threshold does not improve the redirected T-cell attack toward target cells but may result in antigen-independent T-cell activation.

The Hudecek study (23) evaluated scFv’s of different affinities and CARs with different backbones with respect to their efficacy in redirecting T cells. The CAR with higher binding affinity conferred maximum T-cell activation with respect to cytokine release and proliferation compared to the CAR with lower affinity. The redirected cytolytic activity, however, was nearly the same. Although the study confirms previous observations that increase in affinity does not necessarily improve all T-cell effector functions, the comparison of the CARs is alleviated in that two binding domains targeting different epitopes, although in the same domain of the targeted ROR1 molecule, were used.

A recent study explored the situation for TCR modified T cells to determine the affinity threshold with respect to the optimal balance between anti-tumor efficacy and auto-immunity (24). Similar as for CAR modified T cells, TCR redirected anti-tumor activity shows a plateau at a defined TCR affinity, likely due to diminished contribution of TCR affinity to avidity above the threshold. Additional differences probably lie in the ability of different affinity interactions eliciting different effector functions at different antigen concentrations. The observations are in accordance to the CAR situation and strongly suggest that a relatively low affinity threshold is mandatory to avoid self-damage, that high affinity TCRs do not necessarily improve efficacy given the close relationship between anti-tumor activity and auto-immunity.

## The Position of the Targeted Epitope Matters: Membrane Proximal vs. Distal Epitopes as Targets for CAR Engineered T Cells

By using an antibody for targeting, CAR engineered T cells can be redirected toward a variety of epitopes of the same antigen as far as the epitope is accessible to the respective antibody. The various epitopes of a given membrane-bound molecule, however, are not equally good targets for efficient T-cell activation. This was shown when membrane distal and proximal epitopes of the same membrane-bound molecule were targeted by CARs. For instance, when targeting carcinoembryonic antigen (CEA) expressed on gastrointestinal carcinoma cells, a higher degree of T-cell activation was obtained when epitopes closer to the cell membrane were targeted (25). The epitope itself is not the cause of the phenomenon since the isolated, solid phase bound CEA induces T-cell activation independently of the epitope position but dependent of the antibody binding affinity. The distal epitope when expressed in a more membrane proximal position activated CAR T cells with higher efficiency than in the distal position indicating that the position effect of the targeted epitope has, at least in this example, prominent impact on T-cell activation. The accessibility of the epitope for binding additionally impacts the efficiency in CAR-mediated T-cell activation; in the case of CEA targeting, however, the epitope accessibility seems not to be limiting because the distal epitope, which is thought to be more accessible than the more proximal epitope, is superior in binding but less capable in mediating CAR activation. This is in accordance with another report which analyzed the impact of the position of the target epitope on the structural requirements of the CAR (26). Basically the same observation was made when targeting B-cell lymphoma associated CD22 by CAR T cells (27, 28). To explain the observation, a kinetic-segregation model, initially proposed by Davis and van der Merwe (29) and hypothesized also to occur in CAR engineered T cells, is currently favored. The model suggests that targeting membrane distal epitopes increases the size of the CAR-ligand clusters, which in turn permits large phosphatase molecules such as CD45 to enter the synapse and to repress TCR signaling which is less the case when targeting the membrane proximal epitope. The model, however, does not exclude that accessibility and flexibility of the targeted epitope itself may also contribute to some extent.

The best suitable target epitope and binding affinity for optimal CAR T-cell activation remains so far to be empirically evaluated in each case. This is of major relevance given the broad variety of potential targets for a CAR in contrast to the TCR, the specificity of which is restricted to MHC presented peptides.

## CD28 Costimulation Provided by Second Generation CARs: Major Differences to Stimulation Through APCs

First generation CARs provide exclusively only one signaling domain such as CD3ζ-, ϵ-, or FcϵRI γ-chain to initiate redirected activation of pre-stimulated T cells upon CAR binding with antigen. To prevent engineered T cells undergoing activation-induced cell death and anergy, CD28 costimulation simultaneously to CD3ζ signaling is required. CD28 is the prototype of a family of costimulatory molecules that is physiologically engaged on T cells by binding to the respective ligands on antigen-presenting cells (APCs). The agonistic CD28 ligands B7.1 (CD80) and B7.2 (CD86), physiologically expressed on APCs, are missing on most cancer cells with the consequence that the CD3ζ CAR upon binding to cancer cells does not provide the costimulation required for full activation. The limitation was overcome by linking the intracellular signaling domain of CD28 to CD3ζ in one polypeptide chain of the same CAR (30–33). In this so-called “second generation” CAR the artificial fusion of the CD28 and CD3ζ signaling domains facilitates Lck-mediated CD28 phosphorylation that binds and activates phosphatidylinositol 3-kinase for downstream signaling, resulting in full T-cell activation and IL-2 release. Other costimulatory molecules of the TNF-receptor family including 4-1BB (CD137) and OX40 (CD134) can also be integrated into the same CD3ζ CAR molecule or combined with CD28 in a “third generation” CAR. This type of CAR has the advantage that T-cell costimulation occurs in an APC-independent fashion and is accompanied by suppressing inhibitory and/or strengthening stimulatory signals, each costimulatory signal modulating the T-cell effector function in a specific fashion (34). CD28 costimulation is integrated into most currently used CARs because CD28 sustains survival and prolongs polyclonal expansion of engineered T cells without the need of B7–CD28 engagement (35). CD28 co-signaling induces IL-2 that is used in an autocrine fashion by redirected T cells to increase their amplification (36). CD28-CD3ζ CAR signaling moreover counteracts transforming growth factor-β1 (TGF-β1)-mediated repression in T-cell amplification (37). Both prevention from AICD and increased amplification produce prolonged T-cell persistence and an improved anti-tumor attack. Other beneficial properties and some draw backs were recently discussed in more detail (38). Taking advantage of CD28 of other costimulatory moieties like 4-1BB, second generation CARs are currently being explored in early phase clinical trials.

The impact of CAR provided CD28 costimulation on the threshold of antigen-dependent, APC-independent T-cell activation was addressed by using a panel of CARs targeting T cells in the absence of agonistic CD28 ligands (39). CAR provided CD28 costimulation increases cytokine secretion but does not impact the activation threshold or “affinity ceiling,” above which an increase in affinity does not increase T-cell activation. CD28 did not increase sensitivity toward target cells with intermediate or low densities of the respective target antigen. Additional CD28–B7 engagement did not further alter CD28-CD3ζ CAR-mediated T-cell activation. In the presence of a CD3ζ CAR, however, B7 engagement increased IFN-γ secretion indicating that the physiological CD28 costimulation through APCs cooperates with CAR-driven T-cell activation.

Another aspect concerns the fact that most target antigens for adoptive immunotherapy are not exclusively expressed on tumor cells but broadly present on a variety of healthy tissues, although frequently at lower levels. Since CAR provided CD28 costimulation in the absence of APCs does not alter the activation threshold, costimulation does not impact the selectivity of a redirected T-cell attack in peripheral tissues, does not lower the affinity ceiling and antigen-dependent threshold of CAR redirected T cells and thereby protects healthy cells with physiological levels of antigen from a T-cell attack.

With respect to costimulation, there are some fundamental differences in the physiologic vs. CAR-mediated T-cell activation. To induce full T-cell activation, the peptide loaded MHC has to interact with the TCR in a form that allows the appropriate synapse formation on the T-cell and to recruit costimulatory molecules which increase stability during early stages in this process. CD28 recruitment by B7 engagement on APCs sustains formation of the immunological synapse which is accompanied by lower amounts of antigen required for T-cell activation. During T-cell–APC interactions, in particular during early activation events, CD28–B7 binding potentiates synapse formation by increasing the density of the synapse components through approximation of the interacting membranes (40); increased clustering integrates the TCR with costimulatory signaling which can compensate for weak TCR signals (41). The TCR binding threshold exhibits a sharp cutoff between full T-cell activity and no activity; the activation efficiency correlates with the TCR binding to the cognate peptide–MHC on APCs (42). Optimal CD28 costimulation occurs upon high-avidity engagement of dimeric B7.1, followed by dimer dissociation, CD28 down-regulation, and B7.1 internalization (43). CD28-B7 interactions with APCs sustain synapse formation which facilitates T-cell signaling upon low affinity target engagement depending on the extend of supra-molecular clustering (44). This mechanism is in contrast to CD28 CAR-mediated T-cell activation, in particular, the avidity of CAR binding is generally higher than of physiological TCR–MHC interactions. Whether the CAR synapse is formed in the same way as the TCR recruits additional components is so far not resolved. There are, however, some cooperative interactions between the CAR and downstream signaling molecules since additional B7.1-CD28 costimulation improves cytokine secretion initiated by CAR signaling.

## CAR Based Adoptive Cell Therapy Gained Substantial Success in Recent Early Phase Trials

Adoptive cell therapy with CAR engineered T cells is currently being evaluated in a number of early phase trials, some of them are listed in Table 1. Patient’s T cells are modified *ex vivo* by retro- or lenti-viral gene transfer with the respective CAR, amplified to therapeutically relevant numbers and given back to the patient by transfusion. Some of these trials produced encouraging evidence of clinical efficacy. CD19-specific CAR T cells induced complete and lasting remission of refractory CD19^+^ B-cell chronic lymphocytic leukemia (CLL) in all of the first three reported patients (44, 45). When successfully engrafted, CAR T cells expanded *in vivo* more than 1000-fold compared to the initial level, persisted in the peripheral blood and bone marrow for at least 6 months, and continued to express the CAR. T cells were effective in an anti-tumor response even at low dosage levels of about 1.5 × 10^5^ cells/kg (45). The prolonged persistence of CD28-4-1BB-CD3ζ CAR modified T cells is probably due to two effects, the cooperation of costimulation in sustaining T-cell survival in the long-term and the repetitive re-stimulation by CD19^+^ healthy B cells and their progenitors which are also targets for the anti-CD19 CAR T cells. Apart from grade-3 tumor lysis syndrome and a cytokine storm, T-cell infusions had no other acute toxic effects in that trial. Interestingly, there was a delayed increase in the pro-inflammatory cytokines IFN-γ and IL-6, which paralleled the clinical symptoms and coincided with the elimination of leukemia cells from the bone marrow. The clinical application of an IL-6 neutralizing antibody, noteworthy, reduced clinical manifestation of the cytokine storm. The same CAR is currently being evaluated in the treatment of pediatric CD19^+^ acute leukemia with spectacular success, however, relapse of CD19^−^ leukemia during therapy was also observed in one case (46). In previous trials, CAR T cells expanded less and objective tumor responses were modest although clearly documented in two out of three patients (47–50).

**Table 1:** **Recent adoptive cell therapy trials using CAR engineered T cells**.

Target antigen	Disease	CAR signaling domain	ClinicalTrial.gov identifier	Clinical center
CD19	B-CLL	CD28-CD3ζ	NCT00466531	MSKCC
CD19	B-ALL	CD28-CD3ζ	NCT01044069	MSKCC
CD19	Leukemia	CD28-CD3ζ	NCT01416974	MSKCC
CD19	Leukemia/lymphoma	CD28-CD3ζ	NCT00924326	NCI
CD19	Leukemia/lymphoma	CD28-CD3ζ	NCT01087294	NCI
CD19	Leukemia/lymphoma	CD28-CD3ζ vs. CD3ζ	NCT00586391	BCM
CD19	B-NHL/CLL	CD28-CD3ζ vs. CD3ζ	NCT00608270	BCM
CD19	Advanced B-NHL/CLL	CD28-CD3ζ vs. CD3ζ	NCT00709033	BCM
CD19	ALL post-HSCT	CD28-CD3ζ	NCT00840853	BCM
CD19	Leukemia/lymphoma	CD137-CD3ζ	NCT01029366	UP
CD19	B-lymphoid malignancies	CD28-CD3ζ	NCT00968760	MDACC
CD19	B-lineage malignancies	CD28-CD3ζ	NCT01362452	MDACC
CD20	Mantle cell lymphoma/indolent B-NHL	CD28-CD137-CD3ζ	NCT00621452	FHCRC
PMSA	Prostate cancer	CD28-CD3ζ	NCT01140373	MSKCC
CEA	Breast cancer	CD28-CD3ζ	NCT00673829	RWMC
CEA	Colorectal cancer	CD28-CD3ζ	NCT00673322	RWMC
Her2/neu	Lung cancer	CD28-CD3ζ	NCT00889954	BCM
Her2/neu	Osteosarcoma	CD28-CD3ζ	NCT00902044	BCM
Her2/neu	Glioblastoma	CD28-CD3ζ	NCT01109095	BCM
Kappa light chain	B-NHL and B-CLL	CD28-CD3ζ vs. CD3ζ	NCT00881920	BCM

*MSKCC, Memorial Sloan-Kettering Cancer Center; NCI, National Cancer Institute; BCM, Baylor College of Medicine; RWMC, Roger Williams Medical Center; UP, University of Pennsylvania; MDACC, M.D. Anderson Cancer Center; FHCRC, Fred Hutchinson Cancer Research Center*.

Despite recent success, two fatal serious adverse events occurred after infusion of CAR T cells, one of which is at least in part contributed to the CAR targeting specificity. “On-target off-organ” activation of the CAR T cells occurred in the NIH trial based on the fact that the targeted Her2/neu (ErbB2) is ubiquitously expressed on healthy tissues (50). The other adverse event after treatment of a CD19^+^ CLL patient with CD28-CD3ζ CAR T cells was attributed to an extravasation of a latent bacterial infection subsequent to lymphodepletion (51). Despite the observed severe adverse events, MHC-independent targeting of cancer cells by CAR modified T cells showed promise in controlling CD19^+^ leukemia in the long-term; currently initiated and future trials will address whether solid cancer lesions will also successfully be targeted and controlled by CAR T cells.

## Conflict of Interest Statement

The authors declare that the research was conducted in the absence of any commercial or financial relationships that could be construed as a potential conflict of interest.

## References

[1] Morgan RA Dudley ME Wunderlich JR Hughes MS Yang JC Sherry RM Cancer regression in patients after transfer of genetically engineered lymphocytes. Science (2006) 314:126–9 10.1126/science.1129003 16946036 PMC2267026

[2] Marr LA Gilham DE Campbell JD Fraser AR Immunology in the clinic review series; focus on cancer: double trouble for tumours: bi-functional and redirected T cells as effective cancer immunotherapies. Clin Exp Immunol (2012) 167:216–25 10.1111/j.1365-2249.2011.04517.x 22235997 PMC3278687

[3] Jorritsma A Gomez-Eerland R Dokter M van de Kasteele W Zoet YM Doxiadis II Selecting highly affine and well-expressed TCRs for gene therapy of melanoma. Blood (2007) 110:3564–72 10.1182/blood-2007-02-075010 17660381

[4] de Witte MA Bendle GM van den Boom MD Coccoris M Schell TD Tevethia SS TCR gene therapy of spontaneous prostate carcinoma requires in vivo T cell activation. J Immunol (2008) 181:2563–71 18684947 10.4049/jimmunol.181.4.2563 PMC2587021

[5] Gross G Gorochov G Waks T Eshhar Z Generation of effector T cells expressing chimeric T cell receptor with antibody type-specificity. Transplant Proc (1989) 2:127–30 2784887

[6] Zhang T Sentman CL Mouse tumor vasculature expresses NKG2D ligands and can be targeted by chimeric NKG2D-modified T cells. J Immunol (2013) 190:2455–63 10.4049/jimmunol.1201314 23355740 PMC3665362

[7] Zhang T Wu MR Sentman CL An NKp30-based chimeric antigen receptor promotes T cell effector functions and antitumor efficacy in vivo. J Immunol (2012) 189:2290–9 10.4049/jimmunol.1103495 22851709 PMC3633481

[8] Wooldridge L Ekeruche-Makinde J van den Berg HA Skowera A Miles JJ Tan MP A single autoimmune T cell receptor recognizes more than a million different peptides. J Biol Chem (2012) 287:1168–77 10.1074/jbc.M111.289488 22102287 PMC3256900

[9] Hombach A Köhler H Rappl G Abken H Human CD4+ T cells lyse target cells via granzyme/perforin upon circumvention of MHC class II restriction by an antibody-like immunoreceptor. J Immunol (2006) 177:5668–75 17015756 10.4049/jimmunol.177.8.5668

[10] Mezzanzanica D Canevari S Mazzoni A Figini M Colnaghi MI Waks T Transfer of chimeric receptor gene made of variable regions of tumor-specific antibody confers anticarbohydrate specificity on T cells. Cancer Gene Ther (1998) 5:401–7 9917095

[11] Hombach A Heuser C Sircar R Tillmann T Diehl V Kruis W T cell targeting of TAG72+ tumor cells by a chimeric receptor with antibody-like specificity for a carbohydrate epitope. Gastroenterology (1997) 113:1163–70 10.1053/gast.1997.v113.pm9322511 9322511

[12] Westwood JA Smyth MJ Teng MW Moeller M Trapani JA Scott AM Adoptive transfer of T cells modified with a humanized chimeric receptor gene inhibits growth of Lewis-Y-expressing tumors in mice. Proc Natl Acad Sci U S A (2005) 102:19051–6 10.1073/pnas.0504312102 16365285 PMC1323148

[13] Stewart-Jones G Wadle A Hombach A Shenderov E Held G Fischer E Rational development of high-affinity T-cell receptor-like antibodies. Proc Natl Acad Sci U S A (2009) 106:5784–8 10.1073/pnas.0901425106 19307587 PMC2667008

[14] Hombach A Heuser C Marquardt T Wieczarkowiecz A Groneck V Pohl C CD4+ T cells engrafted with a recombinant immunoreceptor efficiently lyse target cells in a MHC antigen- and Fas-independent fashion. J Immunol (2001) 167:1090–6 11441120 10.4049/jimmunol.167.2.1090

[15] Moeller M Kershaw MH Cameron R Westwood JA Trapani JA Smyth MJ Sustained antigen-specific antitumor recall response mediated by gene-modified CD4+ T helper-1 and CD8+ T cells. Cancer Res (2007) 67:11428–37 10.1158/0008-5472.CAN-07-1141 18056471

[16] Kreuwel HT Morgan DJ Krahl T Ko A Sarvetnick N Sherman LA Comparing the relative role of perforin/granzyme versus Fas/Fas ligand cytotoxic pathways in CD8+ T cell-mediated insulin-dependent diabetes mellitus. J Immunol (1999) 163:4335–41 10510373

[17] Shresta S Pham CT Thomas DA Graubert TA Ley TL How do cytotoxic lymphocytes kill their targets? Curr Opin Immunol (1998) 10:581–7 10.1016/S0952-7915(98)80227-6 9794837

[18] Graubert TA Di Persio JF Russell JH Ley TJ Perforin/granzyme-dependent and independent mechanisms are both important for the development of graft-versus-host disease after murine bone marrow transplantation. J Clin Invest (1997) 100:904–11 10.1172/JCI119606 9259590 PMC508263

[19] Darcy PK Haynes NM Snook MB Trapani JA Cerruti L Jane SM Redirected perforin-dependent lysis of colon carcinoma by ex vivo genetically engineered CTL. J Immunol (2000) 164:3705–12 10725729 10.4049/jimmunol.164.7.3705

[20] Yasukawa M Ohminami H Arai J Kasahara Y Ishida Y Fujita S Granule exocytosis, and not the Fas/Fas ligand system, is the main pathway of cytotoxicity mediated by alloantigen-specific CD4(+) as well as CD8(+) cytotoxic T lymphocytes in humans. Blood (2000) 95:2352–5 10733506

[21] Kuball J Schmitz FW Voss RH Ferreira EA Engel R Guillaume P Cooperation of human tumor-reactive CD4+ and CD8+ T cells after redirection of their specificity by a high-affinity p53A2.1-specific TCR. Immunity (2005) 22:117–29 10.1016/j.immuni.2004.12.005 15664164

[22] Chmielewski M Hombach A Heuser C Adams GP Abken H T cell activation by antibody-like immunoreceptors: increase in affinity of the scFv domain above threshold does not increase T cell activation against antigen-positive target cells but decreases selectivity. J Immunol (2004) 173:7647–53 15585893 10.4049/jimmunol.173.12.7647

[23] Hudecek M Lupo-Stanghellini MT Kosasih PL Sommermeyer D Jensen MC Rader C Receptor affinity and extracellular domain modifications affect tumor recognition by ROR1-specific chimeric antigen receptor T cells. Clin Cancer Res (2013) 19:3153–64 10.1158/1078-0432.CCR-13-0330 23620405 PMC3804130

[24] Zhong S Malecek K Johnson LA Yu Z Vega-Saenz de Miera E Darvishian F T-cell receptor affinity and avidity defines antitumor response and autoimmunity in T-cell immunotherapy. Proc Natl Acad Sci U S A (2013) 110:6973–8 10.1073/pnas.1221609110 23576742 PMC3637771

[25] Hombach AA Schildgen V Heuser C Finnern R Gilham DE Abken H T cell activation by antibody-like immunoreceptors: the position of the binding epitope within the target molecule determines the efficiency of activation of redirected T cells. J Immunol (2007) 178:4650–7 17372024 10.4049/jimmunol.178.7.4650

[26] Guest RD Hawkins RE Kirillova N Cheadle EJ Arnold J O’Neill A The role of extracellular spacer regions in the optimal design of chimeric immune receptors: evaluation of four different scFvs and antigens. J Immunother (2005) 28:203–11 10.1097/01.cji.0000161397.96582.59 15838376

[27] James SE Greenberg PD Jensen MC Lin Y Wang J Till BG Antigen sensitivity of CD22-specific chimeric TCR is modulated by target epitope distance from the cell membrane. J Immunol (2008) 180:7028–38 18453625 10.4049/jimmunol.180.10.7028 PMC2585549

[28] Till BG Jensen MC Wang J Qian X Gopal AK Maloney DG CD20-specific adoptive immunotherapy for lymphoma using a chimeric antigen receptor with both CD28 and 4-1BB domains: pilot clinical trial results. Blood (2012) 119:3940–50 10.1182/blood-2011-10-387969 22308288 PMC3350361

[29] Davis SJ van der Merwe PA The kinetic-segregation model: TCR triggering and beyond. Nat Immunol (2006) 7:803–9 10.1038/ni1369 16855606

[30] Hombach A Wieczarkowiecz A Marquardt T Heuser C Usai L Pohl C Tumor specific T cell activation by recombinant immunoreceptors: CD3zeta signaling and CD28 costimulation are simultaneously required for efficient IL-2 secretion and can be integrated into one combined CD28/CD3zeta signaling receptor molecule. J Immunol (2001) 167:6123–31 11714771 10.4049/jimmunol.167.11.6123

[31] Thislethwaite F Mansoor W Gilham DE Hawkins RE Engineering T-cells with antibody based chimeric receptors for effective cancer therapy. Curr Opin Mol Ther (2005) 7:48–55 15732529

[32] Finney HM Lawson AD Bebbington CR Weir AN Chimeric receptors providing both primary and costimulatory signaling in T cells from a single gene product. J Immunol (1998) 161:2791–7 9743337

[33] Hombach A Abken H Costimulation tunes tumor-specific activation of redirected T cells in adoptive immunotherapy. Cancer Immunol Immunther (2007) 56:731–7 10.1007/s00262-006-0249-0 17143613 PMC11029842

[34] Hombach AA Abken H Of chimeric antigen receptors and antibodies: OX40 and 41BB costimulation sharpen up T cell-based immunotherapy of cancer. Immunotherapy (2013) 5:677–81 10.2217/imt.13.54 23829616

[35] Beecham EJ Ma Q Ripley R Junghans RP Coupling CD28 co-stimulation to immunoglobulin T-cell receptor molecules: the dynamics of T-cell proliferation and death. J Immunother (2000) 23:631–42 10.1097/00002371-200011000-00004 11186151

[36] Hombach A Sent D Schneider C Heuser C Koch D Pohl C T cell activation by recombinant receptors: CD28 costimulation is required for IL-2 secretion and receptor mediated T cell proliferation but does not affect receptor mediated target cell lysis. Cancer Res (2001) 61:1976–82 11280755

[37] Koehler H Kofler D Hombach A Abken H CD28 costimulation overcomes transforming growth factor-beta-mediated repression of proliferation of redirected human CD4+ and CD8+ T cells in an antitumor cell attack. Cancer Res (2007) 67:2265–73 10.1158/0008-5472.CAN-06-2098 17332357

[38] Hombach AA Holzinger A Abken H The weal and woe of costimulation in the adoptive therapy of cancer with chimeric antigen receptor (CAR)-redirected T cells. Curr Mol Med (2013) 13:1079–88 10.2174/1566524011313070003 23116267

[39] Chmielewski M Hombach AA Abken H CD28 cosignalling does not affect the activation threshold in a chimeric antigen receptor redirected T cell attack. Gene Ther (2011) 18:62–72 10.1038/gt.2010.127 20944680

[40] Wülfing C Sumen C Sjaastad MD Wu LC Dustin ML Davis MM Costimulation and endogenous MHC ligands contribute to T cell recognition. Nat Immunol (2002) 3:42–7 10.1038/ni741 11731799

[41] Purtic B Pitcher LA van Oers NSC Wülfing C T cell receptor (TCR) clustering in the immunological synapse integrates TCR and costimulatory signalling in selected T cells. Proc Natl Acad Sci U S A (2005) 102:2904–9 10.1073/pnas.0406867102 15703298 PMC549456

[42] Holler PD Kranz DM Quantitative analysis of the contribution of TCR/pepMHC affinity and CD8 to T cell activation. Immunity (2003) 18:255–64 10.1016/S1074-7613(03)00019-0 12594952

[43] Bhatia S Sun K Almo SC Nathenson SG Hodes RJ Dynamic equilibrium of B7-1 dimers and monomers differentially affects immunological synapse formation and T cell activation in response to TCR/CD28 stimulation. J Immunol (2010) 184:1821–8 10.4049/jimmunol.0902869 20065109 PMC4088257

[44] Kalos M Levine BL Porter DL Katz S Grupp SA Bagg A T cells with chimeric antigen receptors have potent antitumor effects and can establish memory in patients with advanced leukemia. Sci Transl Med (2011) 3:95ra73 10.1126/scitranslmed.3002842 21832238 PMC3393096

[45] Porter DL Levine BL Kalos M Bagg A June CH Chimeric antigen receptor-modified T cells in chronic lymphoid leukemia. N Engl J Med (2011) 365:725–33 10.1056/NEJMoa1103849 21830940 PMC3387277

[46] Grupp SA Kalos M Barrett D Aplenc R Porter DL Rheingold SR Chimeric antigen receptor-modified T cells for acute lymphoid leukemia. N Engl J Med (2013) 368:1509–18 10.1056/NEJMoa1215134 23527958 PMC4058440

[47] Pule MA Savoldo B Myers GD Rossig C Russell HV Dotti G Virus-specific T cells engineered to coexpress tumor-specific receptors: persistence and antitumor activity in individuals with neuroblastoma. Nat Med (2008) 14:1264–70 10.1038/nm.1882 18978797 PMC2749734

[48] Till BG Jensen MC Wang J Chen EY Wood BL Greisman HA Adoptive immunotherapy for indolent non-Hodgkin lymphoma and mantle cell lymphoma using genetically modified autologous CD20-specific T cells. Blood (2008) 112:2261–71 10.1182/blood-2007-12-128843 18509084 PMC2532803

[49] Kershaw MH Westwood JA Parker LL Wang G Eshhar Z Mavroukakis SA A phase I study on adoptive immunotherapy using gene-modified T cells for ovarian cancer. Clin Cancer Res (2006) 12:6106–15 10.1158/1078-0432.CCR-06-1183 17062687 PMC2154351

[50] Morgan RA Yang JC Kitano M Dudley ME Laurencot CM Rosenberg SA Case report of a serious adverse event following the administration of T cells transduced with a chimeric antigen receptor recognizing ERBB2. Mol Ther (2010) 18:843–51 10.1038/mt.2010.24 20179677 PMC2862534

[51] Brentjens R Yeh R Bernal Y Riviere I Sadelain M Treatment of chronic lymphocytic leukemia with genetically targeted autologous T cells: case report of an unforeseen adverse event in a phase I clinical trial. Mol Ther (2010) 18:666–8 10.1038/mt.2010.31 20357779 PMC2862525

